# 3D Ultrastructural segmentation of human microvasculature reveals new insights in pericyte-endothelial cell interactions

**DOI:** 10.3389/fcell.2026.1788958

**Published:** 2026-05-12

**Authors:** Timothy E. Vanderleest, Joseph F. Arboleda-Velasquez

**Affiliations:** Schepens Eye Research Institute of Mass Eye and Ear and the Department of Ophthalmology at Harvard Medical School, Boston, MA, United States

**Keywords:** 3D reconstruction, 3D U-net, endothelial cells, pericytes, ultrastructure

## Abstract

**Introduction:**

Capillary structure remains incompletely characterized at the nanoscale level. Advances in 3D electron microscopy datasets provide new opportunities to systematically examine microvascular architecture and cellular interactions.

**Methods:**

We performed 3D image segmentation of human cerebral cortex microvasculature using the publicly available H01 Release dataset. This initial analysis focused on descriptive and quantitative characterization of the basement membrane, peg-and-socket cell interactions, and other subcellular features.

**Results:**

We identified several novel structural features. In addition to known bidirectional peg-and-socket connections between pericytes (PCs) and endothelial cells (ECs), we observed similar structures between neighboring ECs and at interfaces where ECs form junctions with themselves. PC pegs showed an unexpected preference for proximity to EC nuclei and were enriched at PC edges. The PC endoplasmic reticulum (ER) frequently contacted the plasma membrane on the lumen-facing surface, particularly at sites corresponding to sockets of EC pegs. We also identified electron-lucent pockets (ELPs) at interfaces between ECs and PCs, as well as within the basement membrane.

**Discussion:**

These findings expand current understanding of capillary ultrastructure by revealing previously unrecognized interactions between vascular cells and novel subcellular features. The observed structural features may have functional implications for signaling, stability, and vascular mechanics, warranting further investigation.

## Introduction

In recent years there has been a rapid growth in the amount of publicly available 3D electron microscopic data to study structural biology of the brain (Bae et al., 2025; Bloss et al., 2018; Lee et al., 2016; Schneider-Mizell et al., 2021; Shapson-Coe et al., 2024). While this large volume of brain image data is extremely important to the field of connectomics, it also provides vascular researchers with a substantial body of work for analysis. There has already been some research using these brain image databases to study the 3D ultrastructure of the microvasculature (Bonney et al., 2022; Grubb, 2023; Ornelas et al., 2021). The dataset known as the H01 Release, which is the focus of this research, is a publicly available 3D serial section scanning electron microscope (ssSEM) image of a ∼1 cubic millimeter volume of the cerebral cortex of the human brain (Shapson-Coe et al., 2024). This dataset is viewable through the web application Neuroglancer (https://h01-dot-neuroglancer-demo.appspot.com), where one can also read voxel x-y-z coordinates and use these coordinates to download subvolumes of the data via Google Cloud.

Capillaries are composed of two different cell types, endothelial cells (ECs) and pericytes (PCs), which are ensheathed in an extracellular matrix known as the basement membrane or basement membrane. ECs are on the inside, and form the vessel lumen, by making tight “S” shaped junctions with themselves and neighboring ECs. PCs situated abluminal to the ECs, have large, rounded somas where their nucleus resides, with long branching processes that wrap around the vessel. PC processes, categorized as primary (longitudinally oriented) and secondary (circumferentially oriented), have been found to take on different patterns depending on where the PC is localized along the capillary network: pre-capillaries have short primary and long secondary processes, capillaries have long primary and short secondary, and post-capillaries have a stellate (or fractal-like) pattern (Dessalles et al., 2021; Grant et al., 2019). At the interface between ECs and PCs there are cytoplasmic projections known as peg-and-socket junctions. While these peg-and-socket junctions go both ways, PC pegs into EC sockets are more common (Bonney et al., 2022).

The team that generated the H01 Release also performed a remarkable amount of image analysis on this data set of the neurons and glial cells. The group conducted a coarse-grained segmentation of the vasculature using AI and performed measurements of the total length of vasculature (22.6 cm), and the numbers of cells, i.e. 4,604 ECs and 3,549 mural cells (Shapson-Coe et al., 2024). The resolution of this dataset is 4 × 4 × 33 nm, making it possible to observe organelles such as nuclei, mitochondria, the endoplasmic reticulum (ER), and many other subcellular features.

Our aim in studying the microvasculature in the HO1 Release was to gain a deeper understanding of the structure of ECs and PCs, how these cells interact with each other, how organelles are spatially organized in the cells, and whether organelle localization is correlated with cell-cell interaction features. To achieve these goals, we performed 3D segmentation using a combination of semi-automated methods and deep learning and developed automated algorithms to measure features of interest.

## Methods

### Selection of capillaries from the dataset

The main criteria for selecting the capillaries used in this study was their orientation. Capillaries that were oriented with their longitudinal axis in the z-direction were selected so that the axial cross-sections had isotropic pixel size (4 nm in both x and y) and to minimize the total image volume containing the capillary (i.e., a diagonally oriented vessel occupies a larger 3D bounding box). Regions that had significant imaging artifacts that persisted for several consecutive z-layers were avoided. It was not uncommon to have image artifacts, but they generally did not persist along the z-direction for more than a single image. The coordinates specifying the bounding boxes of the three vessels used in this study are recorded in Table 1.

**TABLE 1:** Table of X, Y, and Z coordinates for the bounding box of each of the three vessels used in this study. These coordinates can be used to locate the image data in Neuroglancer.

*Voxel coordinates of whole vessels*			
Vessel name	X range	Y range	Z range
V1	249,800–252,000	175,000–177,200	600–1,519
V2	207,300–209,100	161,130–162,930	0–999
V3	329,300–330,700	187,700–189,350	500–1,529

### Building an image segmentation training dataset using active contours

A large part of our initial segmentation was performed using 2D active contours (Chan and Vese, 2001). Active contours require an initial contour (or boundary line between your object of interest and the background) that is ideally near the true contour and automatically adjusts the contour iteratively based on intrinsic properties such as contour curvature, and extrinsic properties from the image such as edges. Active Contours was used on our 3D image volumes by processing the volume sequentially over the 2D z-slices so that the result for slice *z* was used as an initial contour for slice *z + 1*. After each active contours run completed, errors were manually fixed to avoid propagating errors to the next z-slice. Given that the z-resolution was 33 nm, active contours performed well due to the high degree of similarity between adjacent z-slices. The Matlab Image Processing Toolbox function *activecountour* was used with the following input parameters: 60 iterations, the ‘Chan-Vese’ method, ‘Smooth-factor’ set to 2, and ‘Contraction-Bias’ set to zero. A downside of the active contours algorithm is that only one pixel class (or label) could be handled at a time, and the frequency of errors made it time intensive. All manual correction of errors was performed using a custom Matlab interface to draw on the image to generate a mask that would be used to add or remove pixels from the segmentation label matrix.

While we used Matlab for our data annotation work, there are free options such as Webknossos (https://home.webknossos.org/), Dragonfly (https://dragonfly.comet.tech/), or IMOD (https://www.nitrc.org/projects/imod).

### Using the 3D-Unet for semantic segmentation

The most current 3D U-net (Ronneberger et al., 2015) model that we trained uses 45 image volumes for training and four image volumes for validation. Each volume had variable sizes in *x* and *y* and 200 layers in *z*. These volumes come from the three vessels presented in this study (consisting of a total of 2950 frames) along with another short segment of vessel that contains no nuclei (400 frames). While all the ground truth images that we generated were segmented at full resolution (4 nm × 4 nm x 33 nm), we down sampled the data to (8 nm × 8 nm x 33 nm) for training our model. We trained a 3D U-Net for 7-classes including: background/cytosol, basement membrane, lumen, nuclei, ER, mitochondria, and an undetermined class. We used a patch size of [164, 164, 100], 60 patches per image, and a mini batch size of 12. Training data was augmented using random 90-degree rotations, left-right and up-down flipping, and combined flipping and rotating. The training parameters were the following: max epochs = 200, initial learn rate = 0.0005, learn rate drop period = 5, learn rate drop factor = 0.95, and validation frequency = 400. We trained this model on a Dell Precision 5820 Tower with an Intel Core i9-10900X CPU @ 3.70GHz, a single NVIDIA RTX A6000, and 128 GB of RAM.

### Post-processing methodology after the 3D-Unet and label matrix classes

**FIGURE 1:**
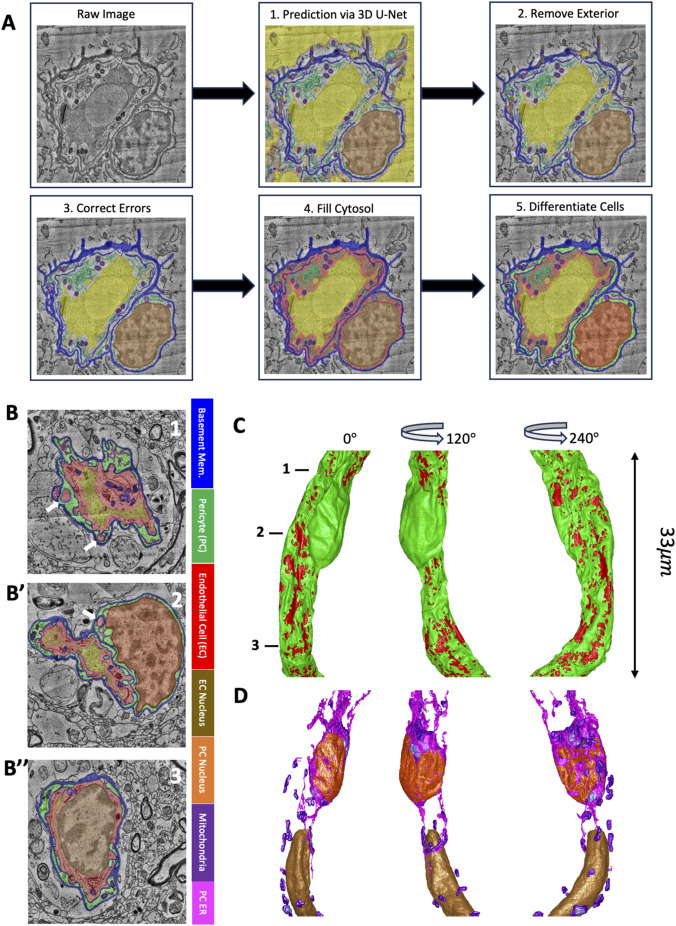
3D Capillary segmentation of the HO1 dataset*.* **(A)** Flow chart of showing the segmentation processing of a raw image. After applying trained 3D U-Net (1) all regions outside the vessel were removed (2), misclassified regions were corrected using automated and manual methods (3), the holes that are cytosol were filled (4), and EC and PC were distinguished based on lumen contact while also using unique organelle labels for the 2 cell types (5). **(B-B’’)** Segmentation color overlay on raw images at three different axial positions of a capillary. Colors indicate basement membrane (blue), PC (green), EC (red), PC nucleus (orange), EC nucleus (brown), mitochondria (purple), PC ER (magenta), and EC endoplasmic reticulum (light green). White arrows point to EC pegs. **(C)** 3D reconstruction of a 33 µm length of capillary showing the EC (red) and PC (green) at three different angles of rotation (0°, 120°, 240°). Numbers indicate axial positions of the corresponding cross-sectional images from B (1), B’ (2), and B’’ (3). **(D)** 3D reconstruction of the same vessel as shown in **(C)** of the EC nucleus (brown), PC nucleus (orange), PC mitochondria (purple), and PC ER (magenta).

The general process used for segmentation (Figure 1A) was to first apply the 3D U-net to predict the classes over the entire cropped volume. The 3D U-Net often predicted mitochondria and ER in the parenchyma but falsely predicted much of the parenchyma as the lumen class. Thus, step 2 was to convert the all the exterior or parenchymal space to the background class. Step 3 involved fixing many of the errors in all the classes but especially focusing on the basement membrane class which forms the boundary of the outer perimeter of the vessel but also forms a boundary between the EC and the PC. Step 3 was critical to step 4, which involved filling the cytosol of the PCs and ECs. Step 5 was to differentiate between the 2 cell types. Determining the cell type was automated for each 2D slice based on whether the region was in contact with the lumen (thus EC) or not (thus PC). After completing these steps, we performed several rounds of manual proofreading. Cell type determination occasionally had errors such as in the case of EC pegs that were not in contact with the lumen in a particular 2D slice (white arrows in Figures 1B-B’’).

The EC-EC junctions are initially classified as basement membrane just like the junctions between ECs and PCs. Therefore, we converted them to their own class to distinguish them. All three types of peg-and-sockets (PC-EC, EC-PC, and EC-EC), which were initially classified as EC or PC cytosol, were also converted to their own classes. In a few rare instances there were mitochondria or ER in pegs, so we assigned new classes to the peg-associated ER and mitochondria so that those classes could be included for accurately measuring pegs volumes and aspect ratio.

The final total number of labels we generated was 26 (integers from 0 to 25), however not all the labels were used. The labels and corresponding cellular structures are the following: (0) background, (1) basement membrane, (2) EC cytosol, (3) PC cytosol, (4) lumen, (5) EC nucleus, (6) PC nucleus, (7) EC ER, (8), PC ER), (9) EC Golgi, (10) PC Golgi, (11) EC mitochondria, (12) PC mitochondria, (13) EC undetermined #1, (14) PC undetermined #1, (15) EC undetermined #2, (16) PC undetermined #2, (17) basement membrane electron lucent pockets, (18) EC-EC junctions, (19) EC peg cytosol, (20) PC peg cytosol, (21) EC peg ER, (22) PC peg ER, (23) EC peg mitochondria, (24) PC peg mitochondria, and (25) EC-EC peg. Our 3D U-Net detects both the ER and Golgi into the ER class because of their similar tubular structures. However, we have not yet completed the task of converting the Golgi to their own labels (9 and 10). The two undetermined classes represent objects in the cytoplasm of both cells: the first being large bubble-like structures (dark edges and more electron lucent centers) that sometimes form large clusters, and the second being large dark spherical and cup-shaped objects. Our trained 3D U-Net grouped both undetermined classes together to reduce the total number of classes.

Upon completing several rounds of searching for errors, using videos to scroll up and down the *z*-axis to identify discontinuities, and correcting them, we would produce 3D reconstructions (Figure 1C) of the cells and the organelles (Figure 1D) as a further quality check on the segmentation. For a video that scrolls through a completed segmentation of a vessel, see Supplementary Video 1.

### Quantification of pericyte coverage

PC coverage was measured as the percentage of the abluminal surface of the EC covered by the PC. The surface of the EC was smoothed by converting PC pegs to the EC mask and EC pegs being removed. The Matlab function *bwperim* was used to get the perimeter of the EC mask and then each perimeter pixel was assigned as being PC-coverage or not based on PC proximity.

### Basement membrane thickness

Basement membrane thickness was measured using a 3D distance transform which accounts for anisotropic voxel size (Mishchenko, 2015). A 3D mask of the PC and EC was used to generate the distance map. We then took the distance values along the outer perimeter voxels of the basement membrane mask. To divide the outer basement membrane mask into sub-domains according to the three classes of non-covered EC, PC processes, and PC soma, distance transforms were used to find the closest of the three regions to the outside basement membrane surface.

### Statistics

For each quantification, except for the peg volume and aspect ratio quantification, the unit of replication was a section of vessel, and the N was three vessels. Each section of vessel had similar lengths (30.36 *μm*, 33 *μm*, and 33.99 *μm* for V1, V2, and V3 respectively), similar cross-sectional areas, a full PC soma, and one EC nucleus (vessel V2’s EC nucleus was partially incomplete). Each quantity of interest, such as the basement membrane thickness over a subdomain of the vessel, was the average voxel value from the entire surface of that subdomain. For the peg volume and aspect ratio quantifications, the pegs of the same type from all three vessels were combined into groups and compared. All statistical tests were performed using GraphPad Prism Version 10.2.2 using a Kruskal–Wallis test.

## Results

### Basic characteristics of three selected vessels

**FIGURE 2:**
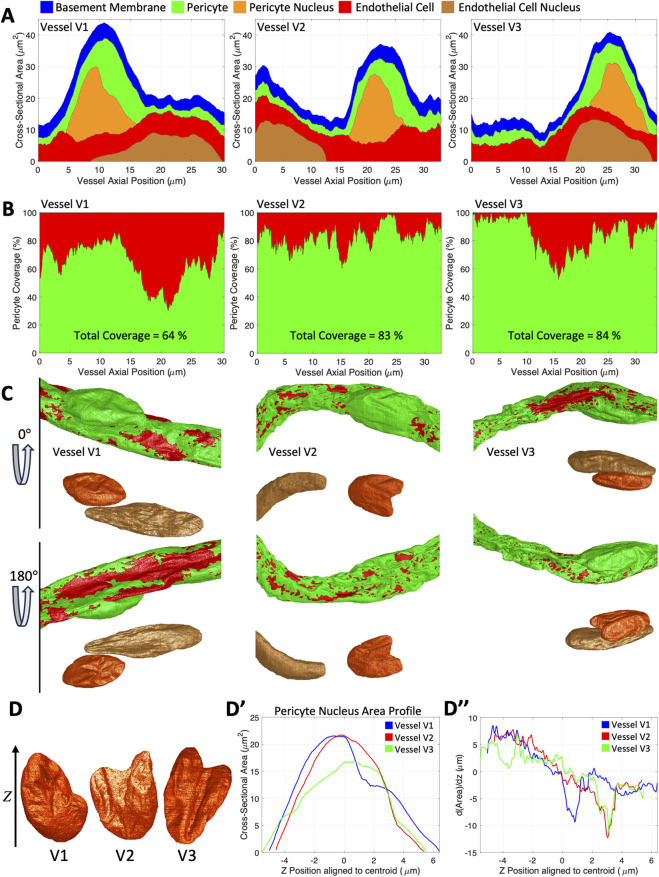
Basic measurements of vessel composition and pericyte coverage. **(A)** Filled area plots, of all three vessels, showing cross-sectional area as a function of axial position of basement membrane (blue), PC (green), EC (red), PC nucleus (orange), and EC nucleus (brown). **(B)** PC coverage percent (green area) as a function of axial position for three different capillary sections. EC nucleus (dashed black line) and pericyte nucleus (solid black line) positions are plotted for reference. **(C)** 3D reconstructions of the vessels (PC in green and EC in red) are shown below at two angles 180° apart (the same color scheme as in panel A was used). **(D)** 3D reconstructions of the three PC nuclei. **(D’)** Axial cross-sectional area profiles of the three PC nuclei colored by vessel number (V1 in blue, V2 in red, and V3 in green). **(D”)** The rate of area change along the axial depth of the three PC nuclei.

To visualize the relative volumes of the cells, basement membrane, and their nuclei location, we generated a stacked area plot showing the class sum of voxels in each x-y cross-section as a function of axial position (Figure 2A). The stacked area plot shows that in each vessel there was one EC nucleus and one PC nucleus with various relative positions. In vessel V2 the EC nucleus was not fully captured in the image volume. Another property of interest was the level of coverage the PC makes with the outer surface of the EC (Figures 2B,C). Vessel V1 had significantly less coverage than vessels V2 and V3 (64% versus 83% and 84% respectively), however there was also significant variability along the z-axis in vessel V3 which drops from near 100%–60% between axial positions 10 and 15 microns. In examining the shapes of the PC nuclei (Figure 2D, Supplementary Video 2), vessels V2 and V3 had a distinct two-lobed morphology resembling a mitten. The area profile along the axial direction (Figure 2D’), after alignment to the centroid, revealed that all three nuclei had an inflection point (or change in curvature) which indicated where one lobe ended and the other continued. A dip in the rate of change of the area (Figure 2D’’) helped pronounce where the inflection points were. In vessels V2 and V3 the inflection points occurred in approximately the same place closer to the end whereas in vessel V1 it occurred closer to the centroid.

### Basement membrane thickness and electron-lucent pocket (ELPs) features

**FIGURE 3:**
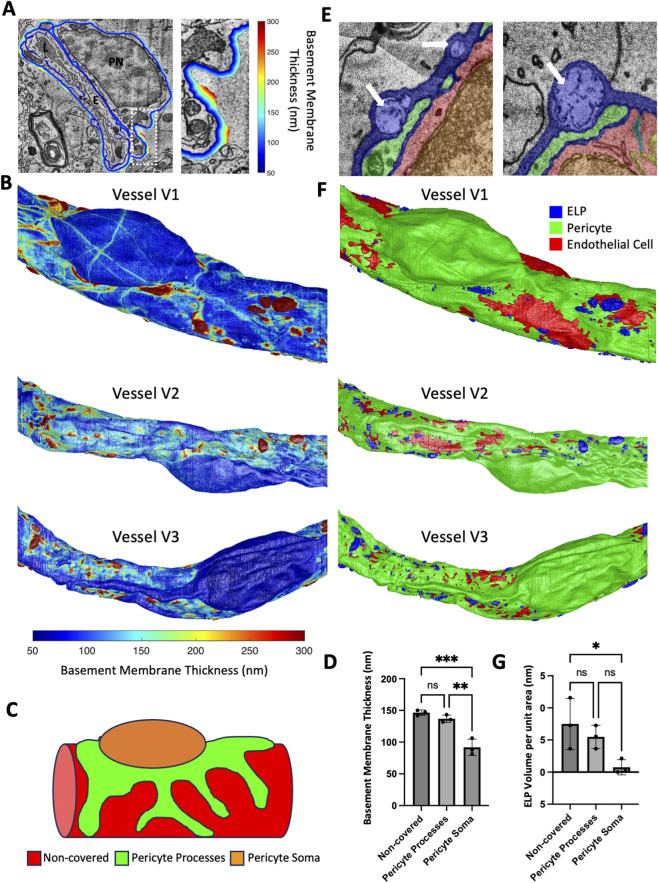
Basement membrane thickness and ELP localization. **(A)** A raw image with a color overlay showing the distance from both PCs and ECs mapped over the basement membrane (left panel). The right panel is a zoom in of the white dashed box in the left panel. **(B)** 3D reconstruction of the outer surface of the basement membrane with a color mapping of the thickness for each of the three vessels. Distance colorbar for all three vessels at the bottom. **(C)** Illustration to show three different domains of the vessel surface for quantification: non-covered (red), PC processes (green), and PC soma (orange). **(D)** Quantification of the basement membrane thickness over three domains. **(E)** Color overlay images showing ELPs in the basement membrane (white arrows). Colors represent basement membrane (blue), PC (green), and EC (red). **(F)** 3D reconstruction of the ELPs (blue), PC (green), and ECs (red) using the same vessels and orientations used in panel **(B,G)** Quantification of the volume of ELPs associated with each domain per unit of surface area of that domain. **(D,G)** N = 3 vessels. Each datapoint is the average over the domain. Mean ± SD error bars shown. Kruskal–Wallis test was performed. * denotes P < 0.05, ** denotes P < 0.01, and *** denotes P < 0.001.

The vascular basement membrane is composed primarily of collagen IV and laminins, that surrounds both ECs and PCs and is crucial for maintaining vascular integrity (Thomsen et al., 2017). To explore how the basement membrane varies over different domains of the vascular surface, we used the 3D segmentation to apply a 3D distance transform from the ECs and PCs to map distance values to the outer surface of the basement membrane (Figure 3A). Mapping distance values to a Jet colormap allowed visualization of the thickness of the basement membrane in 3D (Figure 3B, Supplementary Video 3). The most notable features of the basement membrane were that the region over the PC soma was uniformly thin, and the remainder of the surface was relatively thicker and had numerous outwardly directed bulging spots (Figure 3B). Quantification of the average thickness of the basement membrane over three distinct domains of the surface (PC soma, PC processes, and non-covered; Figure 3C) showed there was no difference between the non-covered EC and the PC process regions, however the PC soma region was significantly thinner (Figure 3D).

Examination of the basement membrane revealed previously unrecognized features that we named electron-lucent pockets (ELPs) which contained dark punctate or filamentous spots (Figure 3E). 3D reconstruction of the ELPs revealed that their locations often coincided with where the basement membrane bulged outward (Figure 3F, Supplementary Video 3). Additionally, we quantified the volume of ELPs over the different vessel surface domains used previously (Figure 3C) and found that there was no difference in the amount of ELP volume per unit surface area between the non-covered EC region and the PC process region (Figure 3G). However, the ELP volume over the PC soma was significantly reduced compared to the non-covered region. While the ELP volume over the PC soma was not significantly different from over the PC processes, this could be due to PC soma positive ELPs at the edge of the PC soma. Besides at the edges, there were no ELPs detected over the PC soma.

### Pericyte pegs contact with endothelial cell nuclei and proximity to pericyte edges

**FIGURE 4:**
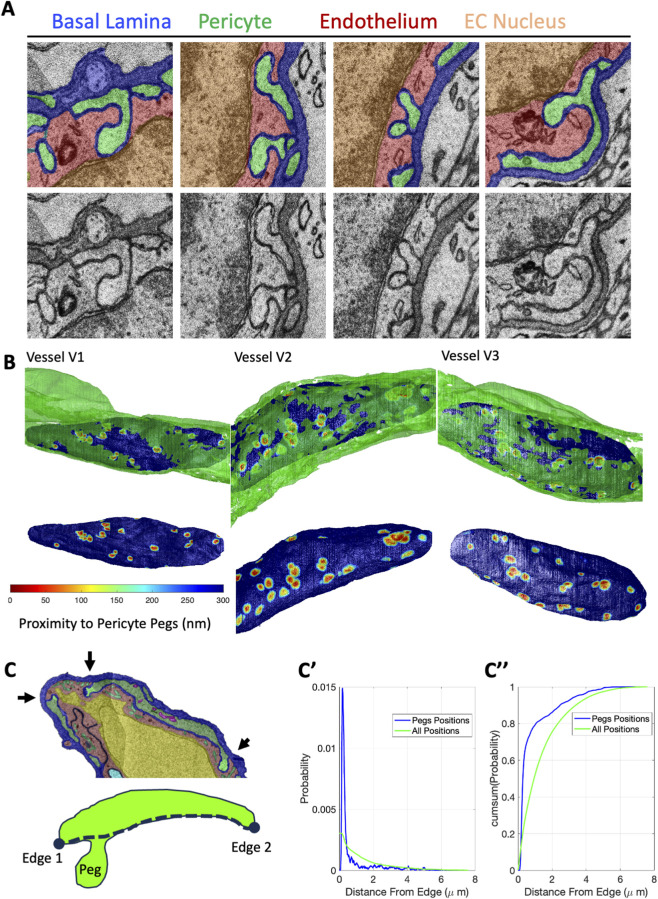
Pericyte pegs interact with the endothelial cell nucleus and are enriched along pericyte edges. **(A)** Four segmentation color-overlay images showing, in X-Y cross sections, PC pegs (green) touching an EC nucleus (orange). Corresponding raw images are shown below each color-overlay panel. **(B)** 3D reconstruction of the PC (green) and the EC nucleus shown with the surface colored according to the proximity of the nearest PC peg voxel (top). The 3D reconstruction of the nucleus in **(B)** but with the PC reconstruction removed (bottom). **(C)** Color overlay image of a vessel with pericyte pegs colored bright green and pointed out with black arrows (top). Illustration of an X-Y cross-section of a PC with a peg. The dashed line on the lumen-facing surface indicates the coordinate system for measuring peg position relative to the nearest edge. **(C′)** Probability normalized distribution of the peg distances from edges for all the PC pegs amongst all three vessels (blue line) and the distribution of all available positions (green line). **(C’’)** Cumulative probability distribution of the probabilities in **(C′)**. **(C’-C”)** N = 3 vessels.

We observed that PC pegs frequently came in close proximity to the EC nucleus (Figure 4A). In many cases there was no visible EC cytoplasm between the PC peg and the EC nucleus. Using a distance transform to map the proximity of PC pegs to the surface of the EC nucleus, we generated a 3D reconstruction of the EC nucleus colored according to this proximity (Figure 4B, Supplementary Video 4). This 3D reconstruction heatmap revealed numerous contact sites on the side of the EC nucleus facing away from the lumen.

PC pegs often appeared near the edges of the PC (Figure 4C). To quantify the frequency, we generated a coordinate system on the lumen-facing surface of each PC section that measures the distance from the nearest edge (Figure 4C). For the pegs in all three vessels, the distribution of positions had a large peak near zero whereas the distribution of all available positions had more of an exponential decay profile (Figure 4C’). The cumulative sum of the probability distribution revealed that 70% of all pegs were withing 0.5 microns of an edge and 80% were within 1 micron of an edge.

### Peg-and-socket junctions between endothelial cells

**FIGURE 5:**
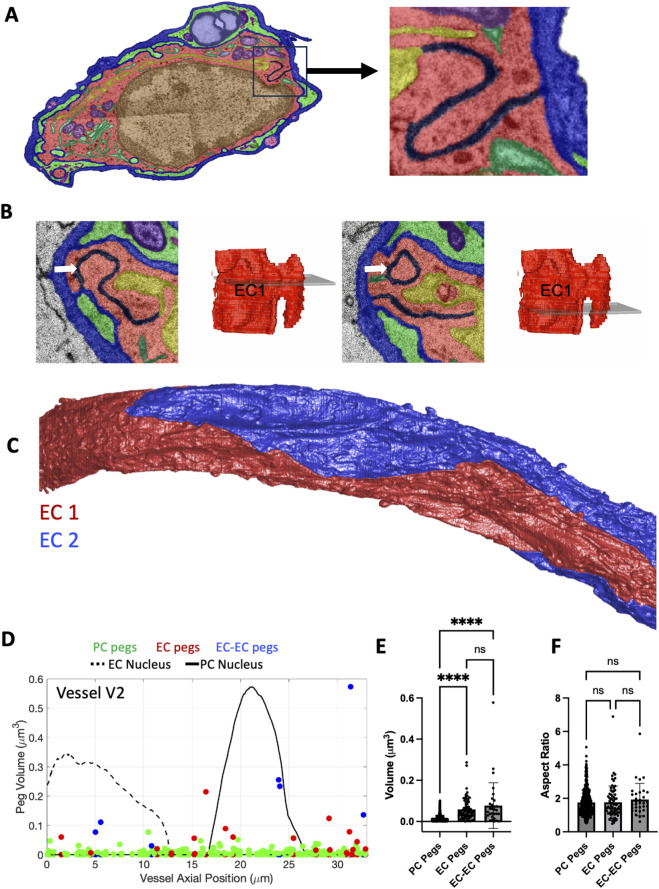
Endothelial cells interact with each other via peg-and-socket junctions. **(A)** Color-overlay image showing the “S” shaped junction between ECs (black). Inset on the right shows a zoomed-in view of the junction. **(B)** Two X-Y cross-sections of the same EC-EC peg with 3D reconstructions and transparent gray plane indicating the positions of the X-Y cross-sections. The EC1 peg is indicated by the white arrow. **(C)** 3D reconstruction of two different ECs (EC1 in red and EC2 in blue) in a section of capillary. **(D)** Scatter plot of peg volumes versus axial position of the three different types of pegs: PC pegs in EC sockets (green), EC pegs in PC sockets (red), and EC pegs in EC sockets (blue). **(E)** Quantification of peg volumes. **(F)** Quantification of the peg aspect ratio, defined as the *z*-axis length divided by the maximum diameter in *x-y*. **(E,F)** N = 32 EC-EC pegs, N = 70 EC-PC pegs, and N = 442 EC-PC pegs. Mean ± SD error bars shown. Kruskal–Wallis test was performed. **** denotes P < 0.0001.

ECs form tight junctions within a single EC as well as with neighboring ECs. In an axial cross-section of a vessel this junction often has a “S”-shape, like locking puzzle pieces (Figure 5A). However, exploration of these junctions in 3D revealed what appears to be peg-and-socket junctions at the interface between ECs (EC-EC peg-and-sockets; Figure 5B). In a 33 µm section of capillary from vessel V2, containing 2 ECs (Figure 5C), we observed 7 EC-EC peg-and-socket junctions between the 2 cells at various axial positions across the vessel (Figure 5D). Vessel V3 only contained one EC with no junction with itself and therefore had no EC-EC pegs. Interestingly, vessel V1 had an elevated frequency of EC-EC pegs (n = 29) but a reduced number of EC-PC pegs (n = 6). Taken together, the EC-EC pegs had similar volumes to the EC-PC pegs, and these were significantly larger than PC-EC pegs (Figure 5E). All three types of pegs were elongated along the Z-axis by on average a factor of just under 2 (Figure 5F).

### Pericyte ER preferentially localizes to the sockets of endothelial pegs

**FIGURE 6:**
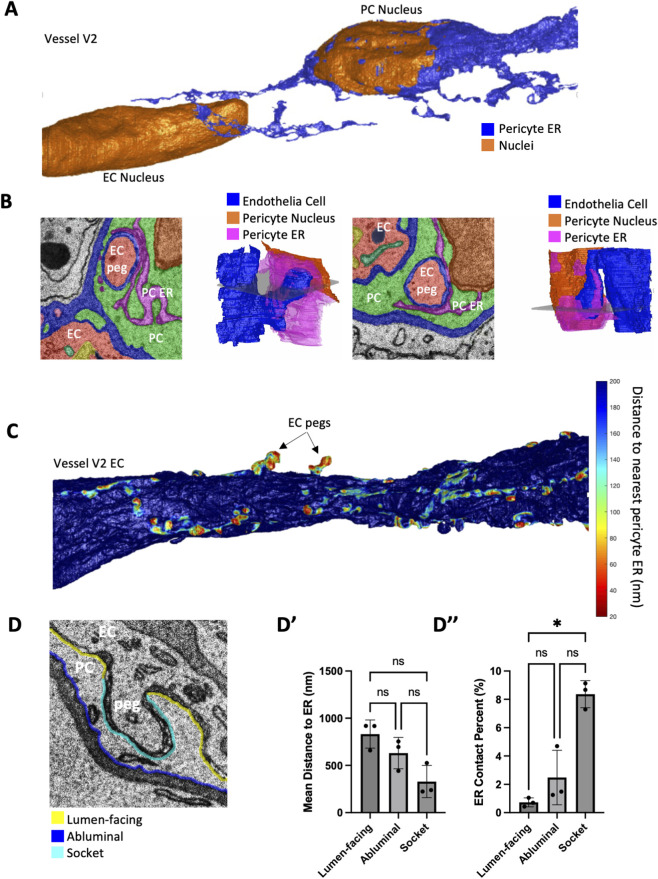
Pericyte endoplasmic reticulum localizes to pericyte sockets of endothelial cell pegs. **(A)** 3D reconstruction of the nuclei (orange) and the PC endoplasmic reticulum (blue). **(B)** Color-overlay images of EC pegs (red) and corresponding 3D reconstructions of the EC pegs (blue), the PC ER (magenta), and the PC nucleus (orange). **(C)** 3D reconstruction of the endothelial surface colored according to the proximity of the nearest PC ER voxel. **(D)** Example image showing the three categories of PC surface used to quantify ER association: Lumen-facing (yellow), abluminal (blue), and socket (cyan). The image has white labels showing the PC, the EC, and the EC peg (peg). **(D′)** Quantification of the average distance to the nearest pericyte ER for the three surfaces. **(D”)** Quantification of the percent of direct contact with the PC ER on each surface. **(D′,D”)** N = 3 vessels. Each datapoint is the average over all voxels of the surface domain. Mean ± SD error bars shown. Kruskal–Wallis test was performed. * denotes P < 0.05.

We also segmented the endoplasmic reticulum (ER) of both the PCs and ECs. The PC ER formed a continuous network of tubules that extended far from the PC nucleus along PC processes (Figure 6A). We also found that the PC ER frequently interacted with the plasma membrane both on the luminal and abluminal side of the PC. One striking observation was that ER appeared to have more frequent contact with the plasma membrane of sockets from EC pegs (Figure 6B). To visualize this in 3D we employed the same technique that we used to visualize the proximity of PC pegs to the EC nucleus, but in this case to visualize the proximity of PC ER to the EC surface (Figure 6C). To determine whether there was a preferential localization for different surfaces of the plasma membrane we segmented the surface into three categories: 1) the side facing the lumen (Lumen-facing), 2) the abluminal side, and 3) the surface of sockets (Figure 6D). Along these three classes of surfaces, we quantified the average distance to the nearest PC ER (Figure 6D’) and the percent of the surface in direct contact with the ER (Figure 6D’’). We found that the PC ER has a significantly higher contact percent with EC socket surfaces compared with the lumen-facing surface.

### ELPs at the interface between pericytes and endothelial cells

**FIGURE 7:**
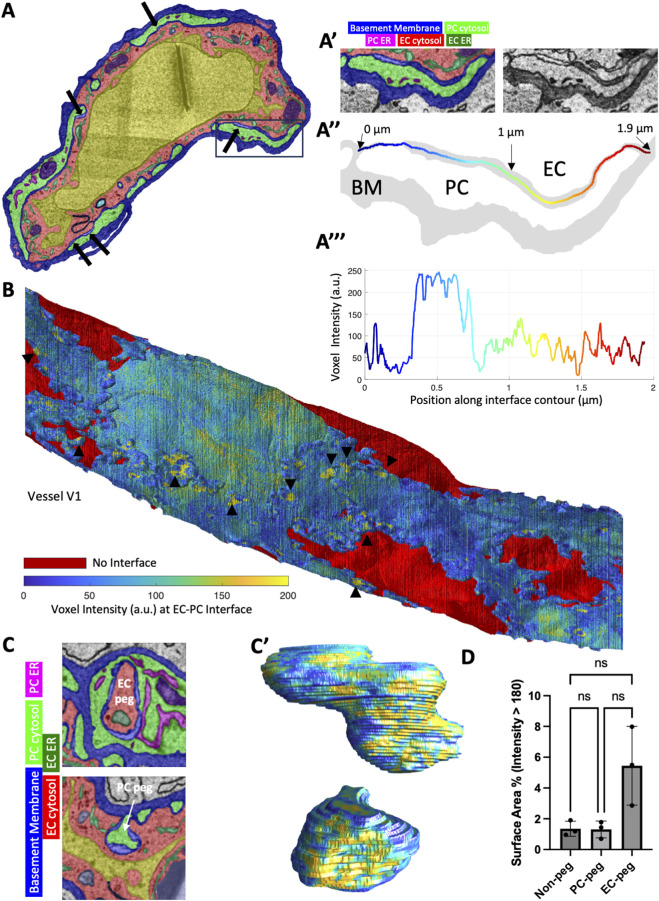
Gap features at the interface between pericytes and endothelial cells. **(A)** Vessel cross-section color overlay image. Black arrows point to regions at the PC (green)-EC (red) interface (blue) that displays “gap features.” **(A′)** Zoom-in view of the black box in **(A)** showing the gap feature in the color overlay (left) and a raw image (right). **(A”)** The basement membrane mask (gray region) has been skeletonized between the PC and the EC and colored according to contour position along the interface. **(A”)** Plot of the image intensity along the PC-EC interface centerline of the interface in **(A”)** using the same color scheme for position. **(B)** 3D reconstruction of vessel one where the exposed EC is colored red and the PC-EC interface is colored according to the image intensity along the PC-EC interface centerline. Black arrowheads indicate gap feature locations. **(C)** Color overlay of an EC peg (red elongated round region) surrounded by the interface (blue) which contains gap feature (left). 3D reconstruction of the EC peg colored by interface intensity (same colorbar in **(B)**). **(C′)** PC peg (green) with interface gaps and 3D reconstruction at right. **(D)** Quantification of the percentage of surface voxels (i.e., the skeleton voxels) that exceeded an intensity 180 units of the maximum 255 units (8-bit images). **(D)** N = 3 vessels. Each datapoint is the average over all voxels of the interface domain. Mean ± SD error bars shown. Kruskal–Wallis test was performed.

The PCs and ECs were generally separated by a thin layer of basement membrane that occasionally narrowed into solid dark line where the 2 cells presumably form direct cell-cell contacts. Interestingly, we also observed a feature where the interface between the cells broadened with an ELP between the cells (Figures 7A,A’). Using a skeletonization algorithm to find the centerline of the segmented cell-cell interface allowed us to plot the image intensity along the middle of the interface (Figures 7A’’, A’’’), which exhibited notably higher intensities at these ELPs. We used the EC-PC interface skeleton from each 2D cross-section to generate a 3D reconstruction of the interface with the interface intensity mapped onto the surface (Figure 7B). The 3D reconstruction revealed numerous spots or patches across the interface between the cells with no obvious patterns of localization. We also found that these ELPs appeared at the cell-cell interface of peg-and-socket connections, both for EC pegs (Figure 7C) and PC pegs (Figure 7C’). To quantify how frequently ELPs appear on EC peg, PC peg, or non-peg interfaces, for each vessel (N = 3) we measured the percent of the interface voxels that exceeded an intensity of 180 (Figure 7D). While the EC pegs measured at over 5% on average compared to ∼1.3% for non-peg or PC-peg interfaces, this difference was not significant.

## Discussion

In this study we provide an initial descriptive analysis of three human cerebral cortex capillaries from the H01 public dataset (Shapson-Coe et al., 2024). While these three vessels represent a total length of 96.36 μm and total volume of 2.5 μm^3^ (4.73 billion voxels), they are a small subset of the estimated 22.6 cm of vasculature contained within the dataset. Additionally, we introduced selection bias by selecting vessels oriented along the z-axis for having a smaller bounding box (i.e., less image data) and relative ease of identifying structures in isotropic *xy* (4 nm × 4 nm) cross-sections. These selected vessels may not fully represent the broad diversity of cerebral microvasculature in different orientations, at vessel branch points, different classes of pericyte morphology, and different locations in the tissue. In this initial study we were limited in the amount of image segmentation we could perform due to the large amount of manual annotation to correct segmentation errors. Future studies, including much larger quantities of vasculature, will be needed to provide a fuller description of ultrastructural diversity.

Another limitation of this study is that there was no independently annotated ground truth data available to quantify the accuracy of our segmentation. All our annotation was manually validated through a careful iterative process of scrolling up and down the z-stack, viewing segmentation color overlays for discontinuities to find errors, and editing them. We focused special attention to regions involved in our analysis such as basement membrane, pegs, nuclei, and ER. The ER of both cells was the most difficult structure to annotate because of its complex and extensive network of tubules. Some of the ER tubules were very small (in some cases just a single dark spot with no visible lumen) and therefore may have gone undetected. All our annotated data has been made available (see Data Availability Statement) to allow other researchers to modify our segmentation or add new class labels to suit their own needs.

The PC nuclei in this study seemed to consist of two lobes that became apparent on one side of the nucleus along the longitudinal axis. In vessels V2 and V3 the two lobes were more distinctive and had almost a mitten-like appearance. In vessel V1 it was less obvious, but the two lobes became more apparent from a plot of the area and rate of change of area along the longitudinal axis of the vessel. If this is a common shape feature of PC nuclei, this asymmetry along the longitudinal axis of the vessel could indicate that PCs are polarized and, if so, the polarity could be associated with the direction of blood flow. A future test of this hypothesis would be to examine multiple PCs on single vessel branches to see if the PC nuclei all have the same polarity. Perhaps the flow of blood cells through capillaries causes deformations to PC nuclei shape.

The basement membrane is an important component of a healthy microvasculature. In some diseases, such as Alzheimer’s disease and diabetic microangiopathy, basement membrane has been found to undergo thickening (Mancardi et al., 1980; Williamson and Kilo, 1976). In this project we were able to identify a couple of interesting properties of the basement membrane in cortical capillaries: 1) the presence of electron lucent pockets, and 2) differential thickness between the PC soma and the rest of the vessel surface. It is not clear whether these ELPs are physiological fluid pockets associated with the glymphatic system or whether they represent some pathological deposit. If these pockets do represent glymphatic fluid in transit it does seem reasonable that the fluid would avoid the PC soma where the thinner membrane could imply a higher resistance path of flow.

The apparent interaction between PC pegs and the EC nucleus has been reported in previous electron microscopy studies in human cerebral capillaries (Cuevas et al., 1984) and in human skeletal muscle capillaries (Bigler et al., 2016). However, in this study 3D image segmentation revealed that a single EC nucleus can have numerous contacts with PC pegs. Whether there is a functional role for these contacts, such as cell-cell communication through the EC nucleus, is unknown but could be a topic for future experimental studies. Our other finding, that PC pegs were enriched at the edges of PC processes in axial slices is mechanically plausible; if these pegs act as mechanical hooks, their position on opposite edges of the PC would allow them to contract the underlying EC to regulate vessel tone or occlude the lumen.

Peg-and-sockets have been identified as bidirectional interactions, i.e., both PC pegs in EC sockets (PC-EC pegs) and EC pegs in PC sockets (EC-PC pegs) have been observed (Bonney et al., 2022). However, while exploring EC junctions we discovered a third type of peg-and-socket: EC pegs in EC sockets (EC-EC pegs). These pegs were the least frequent of the three types. Amongst all three vessels we observed 32 EC-EC, 70 EC-PC, and 442 PC-EC peg-and-sockets. However, vessel V3 contained no EC-EC peg-and-sockets as it was a “seamless” vessel, i.e., it had one EC and did not form a junction with itself (Lammert and Axnick, 2012). Many of the EC-EC peg-and-sockets that we observed appeared to have a different morphology, with cylindrical shape that protrudes both up- and down-stream along the longitudinal axis. Interestingly, while they were elongated, they were not significantly elongated compared to the other pegs. It was intriguing that vessel V1 had elevated frequency of these EC-EC pegs compared to the EC-PC pegs. We hypothesize that, in vessel V1, reduced PC coverage (64% versus 83%–84% in the other two vessels) is compensated by an increase in EC-EC peg formation, which may serve to reinforce EC junctions under conditions of reduced PC support. In future studies, we aim to perform segmentation across a broader set of vessels exhibiting a wide range of PC coverage to further investigate this relationship.

We first identified the ER by tubular structures that were continuous with the PC nuclear envelope and frequently interacted with mitochondria. As we tracked these tubules in 3D we observed that they formed a continuous network that extended along the processes of the PCs much like the ER found extending along the axons and dendrites of neurons (Yperman and Kuijpers, 2023). We were surprised to find that the PC ER made frequent contact with the plasma membrane at sockets of EC pegs. The amount of ER contact area on sockets was significantly greater when compared to the lumen-facing surface. We hypothesize that this finding could point to a functional role of EC pegs as a means of cell-cell communication or calcium signaling mediated by PC ER.

PCs and ECs form contacts with each other to facilitate intercellular communication such as electrical signaling via gap junctions (Jackson, 2022). In this study we observed an interesting feature at the interface between ECs and PCs: spots where it appears that the 2 cells are separated by an ELP rather than the typical basement membrane. We observed that these features appear not only along the normal circumference of the ECs but also at sites of both EC and PC pegs-and-socket junctions. The gap between the cells was much larger than the ∼3.5 nm associated with gap junctions (Perkins et al., 1997), however we hypothesize that these ELPs could be an alternative site of intercellular signaling between ECs and PCs.

We believe there is potential to learn much more from the remaining vessels in the H01 dataset and other current or potential future public datasets. To segment the remaining vasculature in the H01 dataset there are many future challenges such as improving the automation of vessel segmentation and dealing with vessels of other orientations such as those oriented perpendicular to the z-axis. To segment vessels oriented perpendicular to the z-axis, which have distorted axial cross-sections due to anisotropic voxels, one potential solution could be to resample the data to isotropic resolution and employ *x*- and *y*-axis rotations for data augmentation used to train the model. Improving the automation of the segmentation, we believe is a greater challenge. Our current 3D U-Net model makes many errors such as discontinuous basement membrane (sometimes misclassified as lumen or nucleus), and lumen and nucleus classifications outside the vessel. One direction that could be beneficial are the use of convolutional neural networks that incorporate prior knowledge or anatomical constraints (Liu et al., 2023; Oktay et al., 2018), which could potentially help produce a more continuous basement membrane and avoid small fragments of nucleus or lumen. Exploring these other avenues and increasing the quantity of training data are our primary aims to improve the rate of segmenting additional vessels.

## Data Availability

Matlab codes developed for our analysis pipeline are available through Github (https://github.com/timvanderleest/Vessel-Ultrastructure-Analysis). The annotated image data developed in this study have been made available through the Bioimage Archive (https://www.ebi.ac.uk/bioimage-archive) under accession number S-BIAD3187 (Sarkans et al., 2018). The associated raw image data can be obtained by downloading from Google Cloud Storage using a Python script in the Github repository. The trained 3D U-Net model, which was trained using Matlab Deep Learning Toolbox, has been uploaded to the Matlab File Exchange (https://www.mathworks.com/matlabcentral/fileexchange/183578-trained-3d-u-net-for-a-7-class-microvessel-segmentation). An image showing the 3D U-Net training progress metrics (Accuracy and Loss) is also available in the Github repository.
